# Comprehensive Evaluation of Gait Analysis and Kinematics in Adult Degenerative Scoliosis Using Wearable Motion Capture Technologies

**DOI:** 10.3390/s26113617

**Published:** 2026-06-05

**Authors:** Samet Çıklaçandır, Ibrahim Kaya

**Affiliations:** Department of Biomedical Engineering, Izmir Katip Çelebi University, Izmir 35620, Türkiye; ibrahimkaya21@yahoo.com

**Keywords:** adult degenerative scoliosis, gait asymmetry, gait analysis, inertial measurement unit, motion capture, postural instability, wearable sensors

## Abstract

Background: Traditional gait assessments in adult degenerative scoliosis (ADS) often rely on prohibitively expensive, laboratory-bound optoelectronic systems that lack clinical accessibility. This research aims to independently evaluate both lower limbs using a wearable Inertial Measurement Unit (IMU) system, in contrast to studies that employ a unilateral reference, thereby elucidating the unique bilateral asymmetries and dynamic stability patterns exhibited in ADS. Methods: Gait patterns of 20 ADS patients and 15 healthy controls were analyzed using the Rokoko Smartsuit Pro. Segmental kinematic data were integrated with anthropometric mass distribution models to calculate the total body center of mass (CoM). Spatiotemporal parameters, joint range of motion (RoM), and CoM excursions in three planes were statistically compared between the groups. Results: ADS patients exhibited a cautious gait strategy characterized by significantly reduced step speed, shortened step lengths, and increased step width (p<0.05). Temporal analysis showed prolonged stride, stance, and double support time (p<0.001), while cadence remained comparable to healthy controls. A triple-joint deficit, including hip, knee, and ankle, was identified in the sagittal plane, especially with peak flexion reductions reaching up to 55% in the left knee and 38% in the right knee, highlighting profound functional asymmetry (p<0.001). Additionally, the CoM analysis reflected these stability restrictions, showing increased horizontal excursion and reduced vertical oscillation. Conclusions: Our findings suggest that ADS is associated with distinct, bilateral alterations in the lower limb kinematic chain and notable adaptations in dynamic balance parameters, characterized by a cautious gait strategy and profound sagittal triple-joint asymmetries. These findings highlight the feasibility of full-body wearable IMU technology in capturing objective, bilateral gait alterations, providing a foundational baseline that could complement standard static radiography in future clinical evaluations.

## 1. Introduction

**Figure 1:**
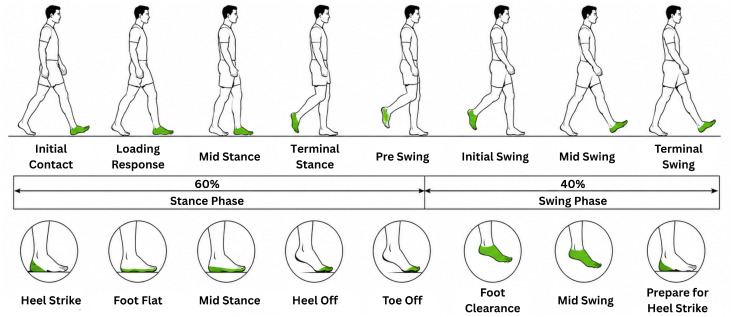
Schematic representation of the gait cycle phases. The cycle is chronologically divided into stance phase components (initial contact, loading response, mid-stance, terminal stance, and pre-swing) and swing phase components (initial swing, mid-swing, and terminal swing), defining the bilateral spatiotemporal boundaries for both groups.

Adult Degenerative Scoliosis (ADS) is a progressive spinal deformity primarily affecting older adults, typically individuals over the age of 50, resulting from asymmetric disc degeneration and facet joint arthropathy [1,2]. This degenerative cascade alters spinal biomechanics, often leading to sagittal and coronal imbalance, significant lower back pain, and postural instability.. In the diagnosis of scoliosis, a physical examination utilizing various imaging techniques, including magnetic resonance imaging (MRIs) and X-rays, a variety of symptoms, and patient medical history are employed [3,4]. Beyond these techniques, gait analysis may be used to gather typical information about an individual’s gait cycle, which can be utilized to diagnose and assess the severity of the condition [5]. Gait is a repeatable, cyclical motion with unique characteristics; its speed and step properties vary from person to person [6]. Figure 1 illustrates the chronological breakdown of the gait cycle phases used to define the bilateral stance and swing duration thresholds. An association with scoliosis can be established by analyzing gait characteristics, postural modifications, muscle activation, and weight distribution [7]. Nevertheless, many neurological and musculoskeletal impairments impact gait and are challenging to distinguish using conventional techniques [8]. For this reason, gait analysis is a beneficial tool in detecting a variety of diseases, including scoliosis. The asymmetric degeneration of intervertebral discs and facet joints in ADS results in a progressive loss of lumbar lordosis, leading to anterior trunk shifting, referred to as sagittal imbalance [2]. To preserve a straight posture and avoid forward falls, patients use lower-limb compensatory mechanisms, such as pelvic retroversion, hip extension, and knee flexion [9]. Chronic structural and postural changes directly modify lower extremity kinematics during locomotion, often resulting in diminished step speed, shortened step length, and limited range of motion in the hip, knee, and ankle joints. Consequently, quantifying these altered mobility patterns provides critical insights into the functional severity of the spinal deformity beyond static radiographic imaging.

Even though gait analysis examinations have numerous established benefits, they are still inaccessible due to their highly advanced technology, high cost, and need for skilled staff [10]. These labs commonly involve electromyography (EMG) equipment, force plates, and high-precision cameras that need to be installed permanently and have drawn-out calibration procedures. Motion capture (MOCAP) cameras are considered the gold standard for gait analysis because of their excellent accuracy [11]. However, advancements in sensor technologies are also getting close to the capabilities provided by optoelectronic systems. Among the many benefits of these Inertial Measurement Unit (IMU) based sensors are their portability, affordability, high degrees of freedom, ease of installation of sensor arrays, simple software, and ability to function in both open and closed spaces with the help of a basic battery system [12]. All of the major moving joints in the human body may be measured using MOCAP suits, which are sensor arrays built and positioned in specific places on a wearable suit. With a very high sampling frequency of 100 Hz, it has been confirmed that these systems, whose calibrations have been successfully completed, are capable of obtaining comprehensive 3D motion data on limb movements [13].

Camera systems are mostly employed in studies concerning gait biomechanics on the ADS. The Vicon camera system with a 200 Hz sample rate was used by Haddas et al. to collect 3D gait motion data from both ADS patients and healthy adults [14]. Kinematic parameters were compared, including gait speed, step length, ankle, knee, pelvic, and trunk ranges of motion, and stance and double support duration associated with the gait cycle. Gait durations were shown to be longer in ADS patients than in healthy people, and it was concluded that gait analysis would offer an objective point of view in clinical assessment. One of the research’s limitations, however, was that just the right side was consulted as a reference; a bilateral study was reportedly required to assess a more profound asymmetry. In another study, Haddas obtained a moderate correlation between ADS and radiographic spinopelvic parameters frequently used by clinicians and gait analysis [15]. Using a motion capture system with four video cameras, Shiba et al. dynamically investigated trunk angle in the sagittal plane [16]. Nonetheless, the research noted that in order to look at the sagittal imbalances brought on by abnormalities, the kinematics of the lower limbs needed to be examined more thoroughly. With the use of a Vicon camera system, Mar et al. determined the Gait Deviation Index in both healthy and symptomatic ADS subjects [17]. ADS patients’ declining gait speed in comparison to the other groups was the reason for the significant correlation with the index based on the kinematic data gathered from both the right and left legs. However, the age gap of almost 20 years between the ADS patients and the healthy group was one of the study’s major limitations. This study also underlined the need for more research to determine the parameters that will reveal the distinctions. The majority of the research, except for this, concentrated on idiopathic scoliosis.

While conventional optoelectronic MOCAP systems remain the gold standard, their high cost and laboratory confinement limit their utility in routine clinical assessments of ADS, where dynamic compensation mechanisms and structural deformities directly affect real-world locomotion. Furthermore, substantial uncertainty remains about whether portable, cost-effective wearable technology can reliably capture the complex bilateral kinematic alterations inherent in ADS gait pathology. To address this gap, this study aimed to quantitatively compare the spatiotemporal and joint kinematic gait parameters of patients with ADS with those of healthy controls using a wearable inertial motion capture system, thereby identifying specific objective biomechanical markers of the disease.

## 2. Materials and Methods

### 2.1. Data Acquisition

**Figure 2:**
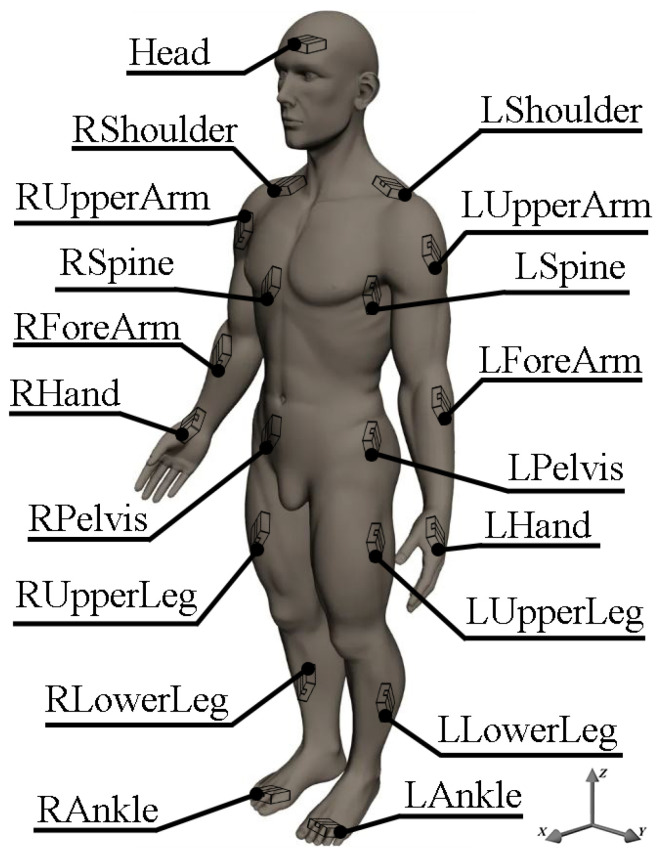
Rokoko Smartsuit Pro Sensor Locations.

Ethical permission was granted by the Izmir Katip Çelebi University Health Research Institutional Review Board committee with the number 0309. All participants in the study were informed of the research’s objective, data collection methodology, and data confidentiality. All procedures were conducted in compliance with the Helsinki Declaration, and signed informed consent was obtained from each participant. As the control group, there were 15 healthy subjects with an average age of 53.70 ± 11.02 who had no complaints, and 20 patients with an average age of 60.10 ± 7.87 who applied to the hospital with ADS concerns. Healthy subjects had an average Body Mass Index (BMI) of 23.34 ± 3.42, and patients with ADS had a BMI of 25.25 ± 2.74. Participants were distributed equally between the genders. In addition, while the slightly higher age profile in the ADS group reflects the natural, age-related degenerative pathogenesis of the condition, both cohorts remained clinically comparable and homogenous in terms of their overall demographic distribution. Specific inclusion criteria were established for each group to ensure clinical validity. For the patient group, individuals were required to have a confirmed clinical and radiological diagnosis of ADS, be aged 40 years or older, be cognitively intact, and have no prior history of spinal or major lower-limb orthopedic surgeries. For the healthy control group, inclusion criteria specified volunteers aged 18 to 75 years who reported no history of scoliosis, spinal deformities, or any musculoskeletal and neurological disorders affecting gait. The ADS cohort presented a mean coronal Cobb angle of 23.8° ± 4.3°, indicating early-to-moderate spinal curvature. In terms of structural deformity localization and directional distribution, a predominant right-sided curve convexity (right-sided apex) was documented across the patient group. Clinically, the presence of chronic back pain was a defining characteristic, with 100% of the included patients reporting persistent low back pain symptomatic for more than six months before the study. Participants were equipped with the Rokoko SmartSuit Pro (v1, Copenhagen, Denmark) to collect movement data. There are 19 IMU sensors in this suit, which are hidden in specific places and connected to the HUB on the back. The sensors also include a 3-axis accelerometer, a 3-axis gyroscope, and a 3-axis magnetometer, which together provide a total of 9 degrees of freedom (DoF) data resolution. Data transmission may occur in the 2.4 MHz or 5 MHz bands of WiFi without requiring a power and data transmission cable, facilitated by the battery pack affixed to the rear of the suit. In addition, the wireless data transmission range of 100 m becomes crucial to prevent data corruption during a gait study. All data can be simultaneously rebuilt on a rigid body using Rokoko Studio (v1.9, Copenhagen, Denmark), the suit’s proprietary software, which transfers data at a sampling rate of 100 frames per second. The suit accommodates individuals with varying BMIs due to its strictly fit and adjustable hook-and-loop fasteners at sensor locations. This suit, manufactured from extremely flexible fabric, may be donned and doffed in under two minutes, preventing participant discomfort. The system’s measurement accuracy and reliability have been confirmed in prior research [13], demonstrating a strong correlation (r>0.99) with gold standard optical motion capture (OMC) modalities. The distribution of sensor locations hosted by the suit is represented in Figure 2.

### 2.2. Testing Protocols

**Figure 3:**
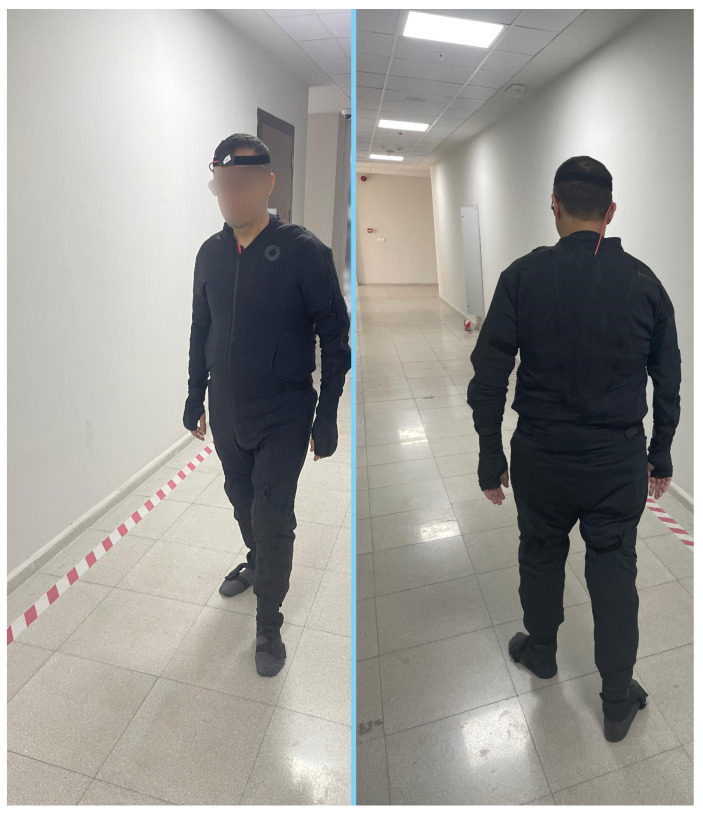
The recording of kinematic data from a patient in a laboratory that was isolated from ferromagnetic materials.

Ferromagnetic items in the surroundings were removed before the recordings to avoid interference with the measurements. The experimental procedure commenced with the same trained technician fitting the wearable suit for each recording to ensure proper alignment of the sensors with the subjects’ anatomical landmarks. To keep the sensors from sliding while moving, hooks and loops were adopted to bind their places on the suit firmly. A 5-min familiarization period was allocated during which participants roamed the laboratory area freely to adjust to the suit’s interface and facilitate natural movement patterns. Following the participants’ confirmation of their relaxation and acclimatization to the wearable equipment, data collection proceeded. For the calibration procedure to be successful, subjects were instructed to remain still in the anatomical reference position for five seconds. The subjects’ anthropometric measurements were taken and transferred to the Rokoko Studio software. Table 1 shows the average values of the anthropometric data of the subjects. In this way, sensor locations were updated, and body segments were arranged in the rigid body diagram. Motion data were collected from the subjects at self-selected speed within a 15 m area isolated from signal noise (Figure 3). The gait protocol was repeated across three continuous trials until a total of seven appropriate gait cycles were obtained per subject within this steady-state area and subsequently averaged. Here, ‘appropriate’ cycles were strictly defined and selected based on the absence of sensor tracking drift, a complete absence of sudden kinematic artifacts or jitter, and clear, identifiable bilateral initial contact events. Cycles affected by transient signal anomalies or visible postural interruptions were rejected.

### 2.3. Data Processing and Kinematic Parameters

To compute the spatiotemporal parameters, the kinematic data was imported into MATLAB (v2025a, Natick, MA, USA). The Rokoko Studio employs a real-time sensor fusion approach to reduce artifacts resulting from magnetic interference and gyroscope drift. This provides the preservation of joint angle accuracy, even during prolonged walking sessions. Notwithstanding, to minimize abrupt motion spikes and high-frequency noise, the data has been processed using a 4th-order Butterworth low-pass filter for digital filtering. To extract the kinematic and spatiotemporal gait parameters, position and orientation data from the lower extremity and trunk sensors were analyzed. Ankle, knee, and hip joint angles were calculated utilizing the 3D orientation data from eight lower extremity sensors, while trunk segments (pelvis, spine, and shoulder sensors) were used to compute multi-planar trunk angles. Spatiotemporal metrics were derived individually for the right and left limbs using the position data from the ankle sensors, mapped directly onto specific gait events (initial contact and toe-off, as illustrated in Figure 1). The temporal segmentation of the gait cycle and event detection (identification of consecutive heel strikes) were performed via manual inspection of the kinematic data. Each initial contact event was visually verified to ensure exact stride segmentation. To capture potential unilateral gait deficits and structural asymmetries, parameters were defined as follows:

Local Step Speed (m/s): Calculated separately for each limb as the ratio of that specific limb’s step distance to the corresponding step time duration (Equation (1)), reflecting the localized propulsion velocity of each lower extremity.

(1) $$\mathrm{Step}\;{\mathrm{Speed}}_{\left(L/R\right)}=\frac{\mathrm{Step}\;{\mathrm{Length}}_{\left(L/R\right)}}{\mathrm{Step}\;{\mathrm{Time}}_{\left(L/R\right)}}$$

Step Length (m): Computed by isolating the coordinate differences exclusively along the progressional X-axis (sagittal plane) between the right ankle (RA) and left ankle (LA) sensors at the moment of initial contact (Equation (2)). (where $t_{IC}$: timestamp of initial contact (IC); $RA_{x},LA_{x}$: *X*-axis coordinates of right and left ankles)

(2) $$\mathrm{Step}\;\mathrm{Length}=\sqrt{{\left(RA_{x}\left(t_{IC}\right)-LA_{x}\left(t_{IC}\right)\right)}^{2}}$$

Step Width (m): Calculated similarly by evaluating the mediolateral distance exclusively along the Y-axis between the RA and LA sensors during the double-support phase (Equation (3)). (where $t_{DS}$: timestamp of double support (DS) phase; $RA_{y},LA_{y}$: *Y*-axis coordinates of right and left ankles)

(3) $$\mathrm{Step}\;\mathrm{Width}=\sqrt{{\left(RA_{y}\left(t_{DS}\right)-LA_{y}\left(t_{DS}\right)\right)}^{2}}$$

Cadence (steps/min): Defined as the total number of steps completed per minute, calculated using the aggregate step durations as formulated in Equation (4).

(4) $$\begin{array}{ccc} Cadence & = & \frac{Step\;Counts}{Time\;(min)} \end{array}$$

Through the use of the position data of all sensors and the segmental mass distribution ratios that are shown in Table 2, it was possible to estimate the location of the body’s center of mass (CoM) while a subject was in motion. The technique of computation that was employed is specified by Equation (5).

(5) $$\begin{array}{ccc} CoM_{\left(x,y,z\right)} & = & \frac{\sum_{m_{i}r_{\left(x,y,z)\right)}}}{\sum_{m_{i}}} \end{array}$$

$m_{i}$ is the mass of the relevant segment and $r_{\left(x,y,z\right)}$ is the location of the relevant sensor in terms of x, y, and z.

To compute the joint ranges of motion (RoM), three sensors are allocated for each anatomical area, and the angle between the generated vectors is represented in Equation (6). The vectors ($v_{1}$ and $v_{2}$) are determined by subtracting the x, y, and z coordinates of the two sensors from one another. The dot products of the vectors are divided by the Euclidean norms in order to arrive at the angle, which is expressed in degrees.

(6) $$\begin{array}{ccc} \Theta^{\circ } & = & \cos^{-1}\left(\frac{\vec{v_{1}}.\vec{v_{2}}}{\left|\vec{v_{1}}\right|\left|\vec{v_{2}}\right|}\right) \end{array}$$

Measurement metrics were represented as mean ± standard deviation (SD). The Shapiro-Wilk test was employed to evaluate the normality of the data distribution, and the Levene test was used to assess the homogeneity of variances (p>0.05). To investigate the main effects of group (ADS vs. healthy control), side (left vs. right), and their interaction, a two-way mixed analysis of variance (ANOVA) was conducted for each bilateral kinematic and spatiotemporal gait parameter. For unilateral parameters without side factors, the Independent Samples t-test was applied. To strictly control the inflation of Type I error rates arising from multiple pairwise comparisons, all post-hoc contrasts were adjusted using the Bonferroni correction method. Accordingly, all post-hoc *p*-values and their corresponding 95% Confidence Intervals (CIs) for the differences were calculated based on this corrected architecture. Effect sizes were quantified using Cohen’s *d* for pairwise group comparisons and partial eta-squared ($\eta_{p}^{2}$) for the ANOVA interaction effects. The alpha level for statistical significance was set at p<0.05. All statistical analyses were conducted using SPSS software (v27, IBM Inc., Chicago, IL, USA).

## 3. Results

The mean and SD measurements of gait spatiotemporal parameters for healthy subjects and patients with ADS are shown in Table 3, comparing these results with those obtained from previous studies. In healthy subjects, consistent with prior studies, cadence and step speed were observed to be elevated, and the periods of the stance and swing phases were also decreased. Likewise, with healthy people, both the step length and the stride length attained longer distances on both legs. In contrast, patients with ADS had a greater step width, which is compatible with the findings shown in Table 3.

Accordingly, a significant difference was observed between ADS patients and healthy subjects regarding step speed, step length, step width, stance time, stride time, double support time, and stride length for both extremities. Contrary to distance-based measurements, temporal factors such as cadence, swing duration, and single support time exhibited no statistically significant differences between the ADS and healthy groups for both the right and left limbs (p>0.05). The comparative analysis indicated an identifiable decline in gait efficiency in the ADS group. In ADS patients, the mean step speed exhibited a 13.3% reduction on the left side and a 19.1% reduction on the right side in comparison to the healthy group. The ADS group demonstrated a 9.8% reduction in step length on the left side and an 8.1% reduction on the right side. Similarly, in terms of stride length for the ADS group a 9.7% decrease was recorded. In contrast, the step width in the ADS group exhibited a 20% increase in step spacing on the left side and a 44.4% increase on the right side. The ADS group revealed a 25% increase on the left side and a 34.2% increase on the right side for stance duration. For stride time, the ADS group had a 19.8% longer duration on the left side and a 26.7% longer duration on the right side. The most notable difference was found in double support time, with the ADS group showing a 67.6% higher support time on the left side and 75.8% higher support time on the right side, markedly different compared to the healthy group. The comparative analysis revealed that gait alterations in the ADS group were more pronounced in the right limb across all spatiotemporal metrics. While both limbs showed significant deviations from the healthy control, the percentage change relative to the healthy group was consistently higher for the right limb, suggesting a non-uniform impact of spinal deformity on bilateral lower limb kinematics.

A two-way mixed ANOVA was executed to evaluate the main effects of Group (ADS vs. healthy), side (left vs. right), and their corresponding group × side interaction effect across the computed gait metrics.The analysis revealed a statistically significant group × side interaction effect for step speed ($p=0.019,\eta_{p}^{2}=0.16$), swing time ($p=0.005,\eta_{p}^{2}=0.21$), and single support time ($p<0.001,\eta_{p}^{2}=0.15$), confirming that the differences between the patient and control groups for these specific metrics varied significantly depending on the limb being evaluated.Conversely, the group × side interaction effect did not reach statistical significance for step length, stance time, stride time, step width, double support time, and cadence (p>0.05 for all comparisons).

Table 4 indicates the mean RoM values for the lower body segments in both the sagittal and coronal planes, as well as their bilateral comparisons and *p*-values. The kinematic study of the sagittal plane demonstrated significant differences in the RoM of the hip and knee joints, along with the left ankle, when contrasting the scoliosis group with the healthy control group. In all segments, the scoliosis group had a consistently reduced RoM compared with the control group (p<0.05). The major decrement limitation was observed in the right knee joint, suggesting that gait may occur with the center of gravity shifted to the right in patients who have spinal deformities. On the other hand, although a reduction in the sagittal plane occurred in the trunk and right ankle, no statistically significant difference was found. In contrast to other joints, the pelvis of a patient with ADS has a modest elevation. The result in patients with scoliosis indicates that although the trunk and right ankle slightly constrain mobility to preserve stability, the pelvic region compensates for this rigidity and the considerable reduction in knee movement by enhancing its mobility, thus proving a biomechanical balancing mechanism.

In the coronal plane, significant differences were observed in the kinematics of the trunk, right knee, and bilateral ankles (p<0.05). The ADS group decreased RoM for the trunk and both ankles, but a substantial increase occurred in the coronal RoM of the right knee. This contrasting pattern may indicate that the restriction of lateral movement in the trunk and ankles is offset by enhanced instability or mobility in the frontal plane at the knee joint level. The most prominent kinematic difference in the coronal plane was detected in the knee joints. The scoliosis group demonstrated a markedly elevated mean RoM in the right knee, with an approximate increase of $8^{\circ }$ relative to the healthy control group (p<0.024). In contrast, although the left knee exhibited a mean increase of $5^{\circ }$, the difference didn’t reach statistical significance (p>0.494). This suggests that frontal plane instability in ADS patients appears asymmetrically, predominantly impacting the right lower limb. These findings demonstrate a significant elevation in coronal plane knee movements throughout the gait cycle in patients with ADS.

Regarding joint kinematics in the sagittal plane, a statistically significant group × side interaction effect was observed for ankle RoM ($p<0.01,\eta_{p}^{2}=0.25$) and hip RoM ($p=0.021,\eta_{p}^{2}=0.14$), demonstrating that sagittal lower-limb restrictions between the groups were highly limb-dependent. In contrast, the interaction effect for sagittal knee RoM did not achieve statistical significance (p>0.05), reflecting a uniform bilateral reduction in total sagittal knee displacement across the ADS cohort. In the coronal plane, a significant group × side interaction effect was selectively captured for knee RoM ($p=0.021,\eta_{p}^{2}=0.16$), underlining an asymmetric mediolateral stabilizing demand at the knee joint level. Conversely, coronal plane interaction effects for hip RoM and ankle RoM were not statistically significant (p>0.05), indicating that coronal modifications in these segments progressed symmetrically across both lower limbs.

**Figure 4:**
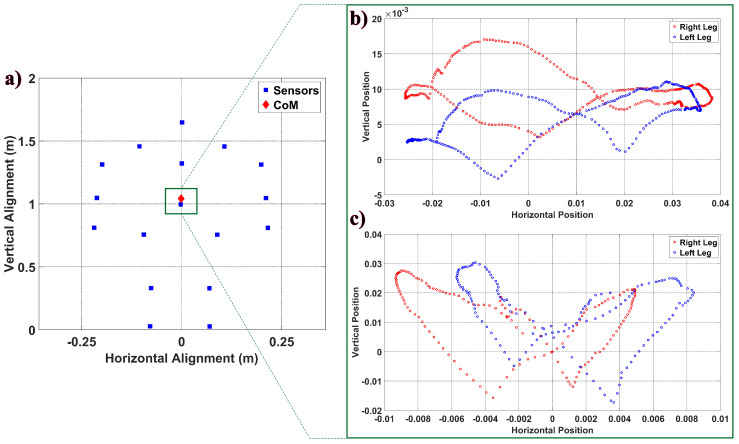
Visualization of computed CoM positions in both horizontal and vertical orientations: (**a**) The positioning of wearable sensors before the beginning of the gait, as well as the coordinates of the determined point of CoM, (**b**) An ADS patient’s shifting CoM according to the coronal plane during gait cycles, (**c**) CoM movement of the healthy subject during the gait cycles.

CoM locations determined throughout the gait, derived from the positions of all segments and their estimated mass values, are illustrated in Figure 4. Using the coronal plane as a reference, Figure 4a shows the initial placements of all sensors together with the approximate location of the center of mass. The X-axis denotes horizontal alignment, whereas the Y-axis indicates vertical alignment in meters. The ambulation of the CoM during a gait cycle is depicted in Figure 4b and Figure 4c, respectively, for a patient with ADS and a healthy subject. The CoM of the ADS patient is highlighted to be more dispersed in the horizontal direction in this graph, which is shown independently for the cycles of the right and left legs. In the patient with ADS, the width of the CoM in the horizontal zone reached roughly 0.06 m, while in the healthy subject, it was measured to be 0.02 m. On the other hand, when it came to vertical directions, the ADS patient occurred in the 0.02 m, but the healthy subject was involved in the 0.05 m area. The results of this demonstrated that the patient with ADS moved in a wider horizontal range and in a more restricted vertical range than the healthy person.

**Figure 5:**
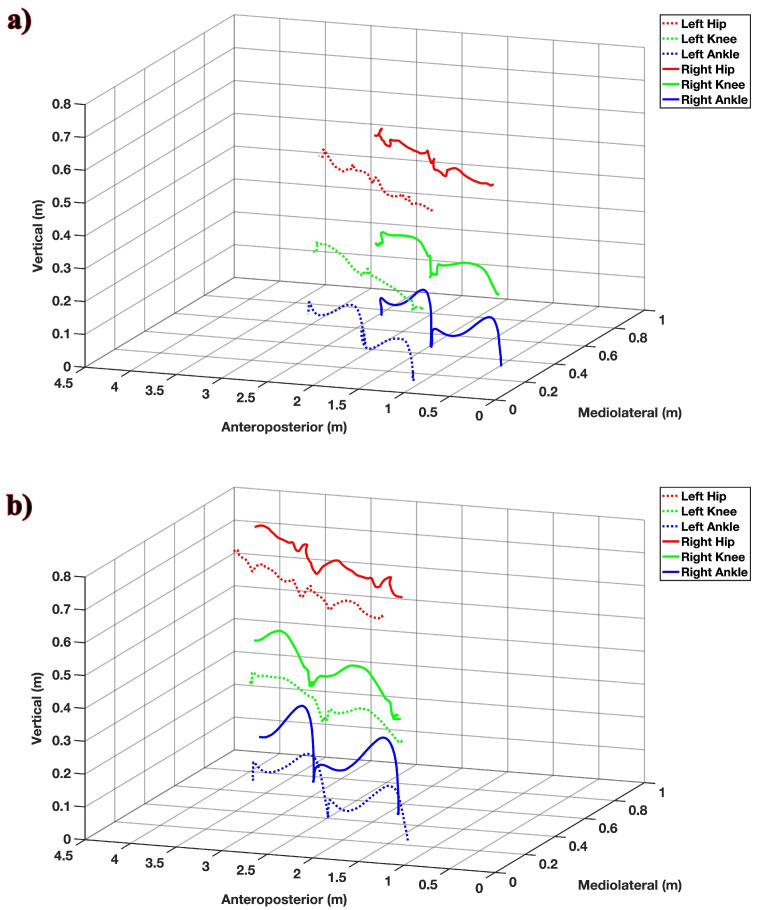
Three-dimensional kinematic trajectories of the hip, knee, and ankle joints tracked over a single complete gait cycle: (**a**) Bilateral 3D trajectories (left and right limbs) obtained from a representative patient with an ADS patient, (**b**) Corresponding bilateral 3D trajectories obtained from a representative healthy control participant. Across both panels, the spatial coordinates and axis scales are synchronized to maintain identical geometric proportions.

The 3D kinematic trajectories tracked over a single complete gait cycle across the hip, knee, and ankle joints visualized distinct spatial differences between the groups (Figure 5). Quantitative assessment of the 3D loops demonstrated that the representative patient with ADS exhibited a restricted vertical excursion across all three tracked joints compared to the healthy control participant. Conversely, the mediolateral distance was visibly increased in the ADS patient, showing a wider horizontal distribution during both stance and swing phases. The spatiotemporal data objectively supported this visual broadening; the ADS group exhibited a significantly greater step width compared to healthy controls on both the left side (mean difference: 0.02 m; p<0.008) and the right side (mean difference: 0.04 m; p<0.001). Furthermore, the anteroposterior displacement was substantially shorter in the ADS trajectory loops. This directly corresponds to the significant reduction observed in the objective step length of the ADS group, with patients demonstrating shorter steps in both left (mean difference: 0.06 m; p<0.038) and right (mean difference: 0.05 m; p=0.014) sides compared to the healthy cohort. In terms of bilateral symmetry, the healthy control participant demonstrated nearly identical, overlapping 3D loops between the left and right sides across all joints (Figure 5a). In contrast, the ADS patient displayed a notable morphological divergence between the left and right trajectories (Figure 5b).

**Figure 6:**
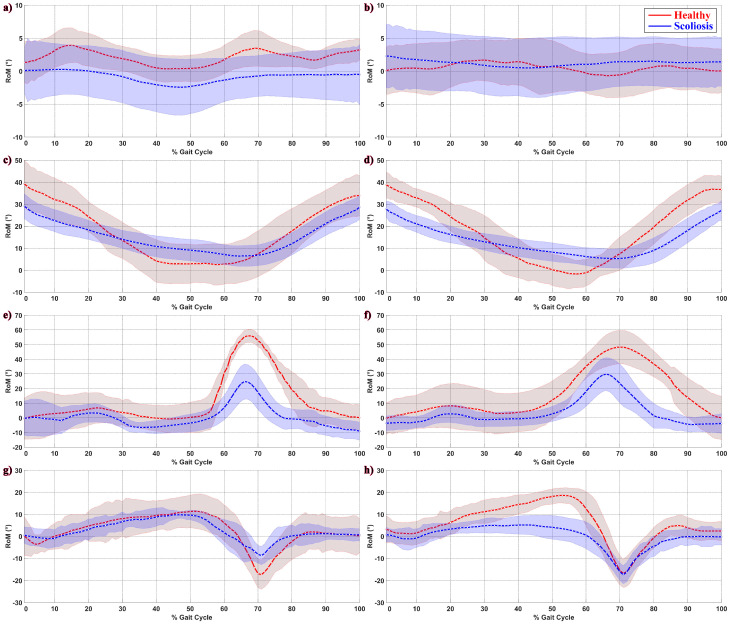
Comparison of lower limb joint kinematics in the sagittal plane between ADS patients and healthy controls over a complete gait cycle. Panels (**a**,**b**) represent left and right pelvic tilt; (**c**,**d**) left and right hip flexion-extension; (**e**,**f**) left and right knee flexion-extension; and (**g**,**h**) left and right ankle dorsi-plantarflexion. The x-axis represents the percentage of the gait cycle (0–100%), and the y-axis indicates the range of motion (RoM) in degrees. Solid lines represent the mean values, and shaded areas indicate the standard deviation (SD).

The kinematics in the sagittal plane (Figure 6) demonstrated substantial differences in the ADS group (blue) relative to the healthy controls (red). Patients with ADS had a limited range of motion (ROM) and a more flexed posture during the gait cycle, particularly in the hip and knee joints (Figure 6c–f). The peak hip flexion-extension angles demonstrated a significant functional decline in the ADS group compared to healthy norms. For the left limb, ADS patients reached a peak of only $22.8^{\circ }\pm 4.6^{\circ }$, which represents a profound 38% reduction from the healthy mean of $36.8^{\circ }\pm 8.5^{\circ }$ (p<0.05). The right limb showed a relatively higher peak of $22.5^{\circ }\pm 4.5^{\circ }$; however, this still constituted a substantial 44.6% decrease relative to the healthy control value of $40.6^{\circ }\pm 6.2^{\circ }$ (p<0.05). These percentage differences highlight that while both hips are severely restricted, the left side undergoes a more aggressive loss of mobility in the sagittal plane. Similarly, a significant reduction was observed in the peak left knee flexion during the swing phase; ADS patients reached only $24.8^{\circ }\pm 4.5^{\circ }$ compared to $55.9^{\circ }\pm 11.9^{\circ }$ in the healthy group, representing a 55.6% decrease (p<0.05). Regarding the right limb, the ADS group exhibited a peak knee flexion of only $29.9^{\circ }\pm 11.6^{\circ }$, whereas the healthy controls reached $48.3^{\circ }\pm 11.4^{\circ }$. This disparity constitutes a 38% reduction (p<0.05). This significant reduction further supports the asymmetrical characteristics of gait disability in people with degenerative scoliosis.

## 4. Discussion and Conclusions

Traditionally, gait analysis in scoliotic populations has depended on gold-standard OMC instrumentation or static radiological evaluations such as computed tomography. Although these technologies provide excellent spatial precision, they are often limited to specialized laboratory environments, need line-of-sight vision, expose the patient to radiation, and are highly expensive. Conversely, IMU-based systems are appropriate for many diagnostic functions owing to their rapid installation, absence of physical area requirements, comprehensive integration with the patient’s body, reproducible and precise data acquisition, and economical nature [13]. This shift toward wearable technology transcends mere convenience; it enables asymmetry mapping, personalized medicine, clinical accessibility, and patient-based elucidation of the kinematic chain [30]. Crucially, the novelty of our work is not merely the use of IMUs, but the paramount significance of this study lies in its demonstration of high-fidelity gait phenotyping within a clinical environment. In addition, different from previous studies that only referenced one extremity side, our major findings are that ADS affects not just the spine but also the entire lower extremities kinematic chain, bilaterally and asymmetrically, in a hierarchical manner, while also revealing the degree of this abnormality.

In optical systems, placing markers on patients is laborious and may result in operator-induced errors or inconsistencies. Conversely, wearable suits include IMUs secretly integrated within the fabric; all sensors are swiftly fastened by tightening straps as a subject dons the suit. The ability to quickly track all body joints also paves the way for advanced kinematic analysis. Another important finding of this study relates to the estimated CoM displacements during gait, modeled through segmental kinematics. While healthy subjects maintain their CoM within a narrow horizontal corridor, patients with ADS exhibit increased horizontal excursion, likely secondary to spinal curvature and pelvic obliquity (Figure 4b). Thus, due to the significant horizontal displacement of the CoM, the patient is compelled to widen their stance to prevent falling (step width increased by 20% on the left and 44.4% on the right). These dynamic observation trends are consistent with static findings by Zabjek et al., who reported reduced stability margins and increased postural oscillation in scoliosis populations, even during a fixed stance [31]. Furthermore, the diminished vertical oscillation documented in the ADS cohort suggests a more constrained ambulatory strategy. Rather than executing the typical sinusoidal vertical translation seen in healthy gait, patients with ADS display a stiffer gait pattern with restricted vertical displacement. This corresponds directly with the documented reductions in hip and knee RoM values, where limited lower-limb joint flexion inherently constrains the vertical trajectory of the body. However, because these CoM parameters are estimated through segmental mass assumptions derived from generic anthropometric models, they must be interpreted as approximate kinematic estimates rather than definitive kinetic evidence of altered dynamic stability.

Furthermore, Haddas et al. demonstrated that patients with ADS exhibit a markedly reduced walking speed in comparison to healthy people. The mean step speed of ADS patients was recorded at 0.78 ± 0.20 m/s, which is 0.27 m/s slower than that of healthy individuals, who averaged 1.05 ± 0.15 m/s [14]. In our study, the average difference in step speed was 0.15 m/s for the left side and 0.22 m/s for the right side, demonstrating that subjects with ADS exhibited slower gaits. Haddas et al. identified a statistically significant decrease in step length and stride length for ADS patients, with mean differences of 0.09 m for step length and 0.19 m for stride length. Similarly, our study detected a difference of approximately 0.06 m for step lengths on both the right and left sides and 0.12 m for stride length. In addition, consistent with Haddas et al.’s study, significant differences were observed in stride, stance, and double-support times, whereas no differences were detected in swing and single-support times. On the other hand, although Haddas et al. observed a statistically significant difference of approximately 13.6 steps per minute between the mean cadences of scoliosis patients and healthy subjects, our analysis revealed no statistically significant difference. The typical cadence values ranged from 109 to 110, and previous studies indicated roughly 110 steps per minute in both individuals with ADS and healthy subjects, with no statistically significant difference [20,26,32]. Moreover, Mahaudens et al. indicated that in patients, stride length was reduced in 7% of cases, and stance phase duration was diminished in 2% of cases with statistical significance [26]. The main findings from gait spatiotemporal parameters reveal that patients with ADS exhibit a reduced step and shorter stride length, alongside increased step width values, indicating an expansion of the base of support to enhance balance. Additionally, the prolonged durations of stance and double support reflect the patient’s endeavor to decrease the time spent on a single leg. One of the main causes for this is that when the patient feels unstable on one leg, they attempt to enhance stability by prolonging the duration that both feet remain on the ground. On the other hand, in cadence, while differences between the groups were roughly comparable, changes in step lengths and speeds within the ADS group were maintained in frequency, yet structurally indicated an unstable gait. The fact that the significantly reduced step speed and shorter stride length in the ADS group corroborate the assertion that the cadence values were similar in both groups.

Regarding RoM, a reduction in trunk angle was noted in the sagittal plane in ADS patients, aligning with prior study [14]; however, this difference lacked adequate statistical significance. In contrast, in the coronal plane, the ADS group exhibited a lower RoM with a difference of 37.5% in mean trunk angle, resulting in a significant difference between the groups. Haddas et al. also found this difference to be 25%, which is statistically significant. In patients with ADS, the spine exhibits reduced flexibility in the coronal plane due to asymmetric loading and degeneration, hence limiting the natural lateral swaying motion, while the dynamic range for forward and backward trunk movement is diminished. Mahaudens et al. similarly determined that patients exhibited a 21% to 28% reduction in pelvic and hip RoM in the coronal plane compared to healthy groups [26]. The substantial decrease for the ADS groups in all three primary joints (hip, knee, and ankle) within the sagittal plane signifies a systemic dysfunction of the lower limb kinematic chain. This study, unlike other research, demonstrated the disparity between the right and left sides, with a more evident global restriction on the left side, indicating a lateralized severity of the kinematic chain collapse (Figure 6).

According to the two-way mixed ANOVA, these findings indicate that the structural spinal asymmetry in ADS forces the lower limbs to execute asymmetric compensatory strategies rather than a uniform global slowing. This limb-dependent kinematic breakdown aligns closely with recent wearable analytics in ADS cohorts [31], where asymmetrical loading apexes across the degenerated spinal curves typically restrict swing-phase transitions on the more affected side while forcing the contralateral limb to adjust local velocity metrics. Conversely, the non-significant interaction effects for total sagittal knee RoM, step width, and double support time (p>0.05) demonstrate a parallel, bilaterally uniform adaptation strategy. Similar to the dynamic stabilization templates observed in asymmetrical neuromuscular cohorts, ADS patients appear to uniformly sacrifice total knee displacement and symmetrically increase their base of support to successfully mitigate the increased horizontal CoM excursions.

The wearable suit improved the determination of specific characteristics in ADS patients by gathering kinematic measurements from all segments. Nevertheless, in comparison to alternative approaches, this system poses several limitations. IMU systems can be subject to various manipulations caused by environmental noise during the data acquisition process. Ferromagnetic components located in the surroundings or on the subject can disrupt the data; thus, it is necessary to remove all metallic objects from both the environment and the patient before measurement. In addition, because of the nature of IMU-based systems, particularly during longer gait tests, a drift in joint angles may arise as a result of integration problems [33]. As segmental mass percentages are derived from a healthy population, a limitation of these approaches is their inability to fully account for the asymmetrical mass distribution resulting from scoliosis. Notwithstanding the intrinsic technological constraints of inertial technology, its high ecological validity and capacity to capture dynamic full-body kinematics in a clinical environment offer a more comprehensive functional assessment of ADS than static radiological imaging [34]. The significant statistical power evident in our combined RoM and CoM excursions indicates that the detected abnormal patterns surpass the system’s noise thresholds. On the other hand, this study analyzed a relatively small patient cohort (*n* = 15). Due to this limited sample size, evaluating a large number of gait variables without strict corrections for multiple comparisons increases the risk of Type I errors. Therefore, marginally significant results close to *p* = 0.05 must be interpreted with caution as preliminary trends that require validation in larger, multi-center cohorts. Moreover, a minor demographic variance was observed between the cohorts, with the ADS group exhibiting an expectedly higher mean age. While these differences reflect the natural clinical epidemiology of patients seeking care for age-related degenerative scoliosis, they introduce a potential confounding risk. Due to sample size constraints, formal statistical covariate adjustments were not executed. Future large-scale trials with strict age- and BMI-matching protocols are warranted to mathematically isolate these minor demographic variances from the wearable-derived gait biomarkers. Additionally, this study lacked concurrent validation against gold-standard optoelectronic systems within this specific cohort. While the Rokoko system’s reliability is supported by prior literature demonstrating strong correlations with optical motion capture [13], simultaneous utilization of both tracking systems in future studies would be highly beneficial to fully complement and formalize the system’s accuracy under severe spinal deformity conditions. Moreover, the lack of Ground Reaction Force (GRF) data from force plates and EMG recordings constrains our capacity to analyze the fundamental kinematics and muscle activation patterns that influence these gait modifications. Future studies that combine kinetic and kinematic data might yield a more comprehensive picture of mechanical work and energy expenditure in ADS patients. Also, this study did not categorize patients according to the severity of spinal deformity or specific curve types, which could affect the extent of gait asymmetry. The diagnostic sensitivity of this approach encourages future investigation into additional neuro-musculoskeletal conditions that affect gait, including Parkinson’s disease and hip-spine syndrome. Prospective investigations are necessary to assess the evolution of these kinematic markers after surgical treatments or personalized rehabilitation protocols. The incorporation of IMU-based gait analysis into standard clinical practice may establish an objective, functional classification system that enhances traditional radiological evaluations in the treatment of ADS. The present study does not use machine learning or artificial intelligence frameworks, yet digital biomarkers still struggle to extract robust information from small pathological cohorts. Recent studies show that multivariate modeling, generative AI, and data balancing may optimize gait classification in limited movement-disorder datasets [35]. IMU-based parameters for adult spinal abnormalities may be more sensitive for diagnosis if large-scale research uses prospective computational paradigms.

## Figures and Tables

**Table 1:** Anthropometric Measurement of Subjects (cm).

Measurements	Scoliosis Patients	Healthy Adults
Height	165.2 ± 11.3	167.8 ± 5.8
Foot Length	21.8 ± 2.3	24.8 ± 1.9
Arm Span	159.3 ± 8.7	162 ± 6.9
Shoulder Height	130.4 ± 7.5	135.4 ± 4.9
Shoulder Width	19.4 ± 2.1	20.4 ± 1.1
Pelvis Height	98.3 ± 4.4	94 ± 5.5
Pelvis Width	27.6 ± 3.7	25.6 ± 2.1
Knee Height	43.6 ± 5.3	47 ± 2.5
Manus Length	41.2 ± 4.8	44.2 ± 2.4

**Table 2:** Distribution of segmental masses in the human body (as a percentage of body mass) [18,19].

Sensors	Left	Right
Upper Legs (UL)	10	10
Lower Legs (LL)	4.5	4.5
Feet (F)	1.3	1.3
Shoulders (S)	6.3	6.3
Upper Arms (UA)	2.7	2.7
Forearms (FA)	1.6	1.6
Hands (HA)	0.6	0.6
Pelvis (P)	20	
Head (HE)	6	
Chest (C)	20	

**Table 3:** Comparison of bilateral spatiotemporal and kinematic gait characteristics of lower limbs between ADS patients and healthy controls, including statistical analysis and benchmarking against prior studies.

	Published Norm		N = 15	N = 20	Post-Hoc p [95% CI] (d)
Left	Healthy (N = 294) [20]	Scoliosis	Healthy	Scoliosis	
Step Speed (m/s)	1.09 ± 0.08	0.8 ± 0.3 (N = 16) [21]	1.13 ± 0.12	0.98 ± 0.15	0.059 [0.01, 0.23] d = 0.83
Cadence (steps/min)	108.76 ± 4.49	110.3 ± 5 (N = 19) [22]	109.64 ± 7.05	111.01 ± 5.18	0.500 [−4.02, 9.33] d = 0.43
Step Length (m)	0.61 ± 0.05	0.69 ± 0.03 (N = 19) [22]	0.61 ± 0.05	0.55 ± 0.06	0.038 * [0.01, 0.12] d = 1.23
Step Width (m)	0.08 ± 0.01	0.09 ± 0.03 (N = 16) [21]	0.1 ± 0.04	0.12 ± 0.02	0.008 [0.01, 0.03] d = 0.86
Stance time (s)	0.72 ± 0.03	0.66 (N = 24) [23]	0.76 ± 0.12	0.95 ± 0.25	0.001 * [0.75, 0.93] d = 2.78
Swing time (s)	0.41 ± 0.01	0.42 (N = 24) [23]	0.4 ± 0.08	0.4 ± 0.24	0.989 [−0.03, 0.04] d = 0.11
Stride Time (s)	1.12 ± 0.04	1.07 (N = 24) [23]	1.16 ± 0.14	1.39 ± 0.23	0.001 * [0.75, 0.91] d = 2.91
Double Support Time (s)	0.31 ± 0.02	0.12 (N = 24) [23]	0.34 ± 0.13	0.57 ± 0.36	0.001 * [0.36, 0.48] d = 1.93
Single Support Time (s)	0.40 ± 0.02	0.48 (N = 24) [23]	0.43 ± 0.05	0.41 ± 0.07	0.245 [−0.02, 0.14] d = 0.28
**Right**	N = 15 [14]	N = 20 [14]			
Step Speed (m/s)	1.05 ± 0.15	0.78 ± 0.20	1.15 ± 0.14	0.93 ± 0.13	0.001 * [0.21, 0.42] d = 2.51
Cadence (steps/min)	106.74 ± 12.05	93.12 ± 10.43	109.55 ± 7.81	110.41 ± 7.98	0.994 [−7.83, 7.47] d = 0.02
Step Length (m)	0.60 ± 0.04	0.51 ± 0.11	0.62 ± 0.06	0.57 ± 0.05	0.014 * [0.03, 0.13] d = 1.47
Step Width (m)	0.10 ± 0.02	0.13 ± 0.02	0.09 ± 0.06	0.13 ± 0.01	0.001 * [0.02, 0.04] d = 2.17
Stance time (s)	0.73 ± 0.07	0.88 ± 0.08	0.73 ± 0.1	0.98 ± 0.24	0.001 * [0.66, 0.82] d = 2.44
Swing time (s)	0.40 ± 0.10	0.42 ± 0.09	0.43 ± 0.05	0.49 ± 0.37	0.201 [−0.01, 0.13] d = 0.27
Stride Time (s)	1.14 ± 0.13	1.31 ± 0.16	1.16 ± 0.13	1.47 ± 0.31	0.001 * [0.73, 0.88] d = 2.61
Double Support Time (s)	0.27 ± 0.07	0.39 ± 0.14	0.33 ± 0.12	0.58 ± 0.37	0.001 * [0.28, 0.38] d = 1.81
Single Support Time (s)	0.43 ± 0.04	0.45 ± 0.07	0.41 ± 0.07	0.4 ± 0.24	0.972 [−0.04, 0.03] d = 0.02
Stride Length (m)	1.18 ± 0.09	0.99 ± 0.19	1.24 ± 0.06	1.12 ± 0.06	0.001 * [0.08, 0.21] d = 2.11

* denotes a statistically significant difference between the groups.

**Table 4:** Bilateral kinematic evaluation of lower limb segments during gait: Mean RoM and SD values throughout the sagittal and coronal planes, accompanied by statistical significance and comparison with previous studies.

Sagittal Plane	Published Norm		N = 15	N = 20	Post-Hoc p [95% CI] (d)
	Healthy	Scoliotic	Healthy	Scoliotic	
Trunk	4.70 ± 2.20 (N = 20) [24]	3.36 ± 0.96 (N = 20) [14]	7.12 ± 0.72	6.84 ± 3.18	0.842 [−2.07, 2.63] d = 0.11
Pelvic	6.53 ± 6.97 (N = 20) [25]	3.00 ± 1.38 (N = 20) [14]	3.99 ± 1.25	5.48 ± 5.59	0.954 [−8.46, 6.31] d = 0.11
Left Hip	42.4 ± 3.3 (N = 13) [26]	40.8 ± 1.8 (N = 12) [26]	41.05 ± 9.99	30.85 ± 4.74	0.072 [0.91, 19.48] d = 1.59
Right Hip	38.23 ± 3.22 (N = 15) [14]	33.17 ± 4.32 (N = 20) [14]	40.89 ± 9.15	30.06 ± 4.65	0.001 * [5.71, 15.95] d = 1.68
Left Knee	62.2 ± 3.8 (N = 13) [26]	55.6 ± 7 (N = 16) [26]	48.83 ± 3.09	36.13 ± 9.24	0.001 * [6.51, 18.16] d = 1.79
Right Knee	46.85 ± 15.39 (N = 15) [14]	32.76 ± 14.64 (N = 20) [14]	47.25 ± 6.41	33.57 ± 6.68	0.001 * [6.94, 20.41] d = 2.03
Left Ankle	31.4 ± 6.1 (N = 13) [26]	26.9 ± 5.7 (N = 12) [26]	31.17 ± 7.82	25.44 ± 8.53	0.099 [0.01, 11.45] d = 0.69
Right Ankle	22.03 ± 4.90 (N = 15) [14]	25.42 ± 6.03 (N = 20) [14]	28.27 ± 6.37	27.26 ± 7.37	0.754 [−3.83, 5.84] d = 0.14
**Coronal Plane**					
Trunk	8.93 ± 3.12 (N = 15) [14]	6.70 ± 2.80 (N = 20) [14]	11.33 ± 4.29	7.08 ± 2.44	0.001 * [1.92, 6.58] d = 1.27
Pelvic	6.01 ± 2.53 (N = 20) [27]	6.2 ± 1.5 (N = 12) [26]	6.7 ± 2.13	8.03 ± 5.02	0.775 [−9.29, 5.37] d = 0.19
Left Hip	13 ±1.7 (N = 13) [26]	10.2 ± 1.8 (N = 12) [26]	13.83 ± 5.67	10.3 ± 5.01	0.128 [−0.14, 7.21] d = 0.67
Right Hip	10.71 ± 3.06 (N = 20) [25]	9.29 ± 1.83 (N = 20) [14]	11.81 ± 3.43	8.81 ± 5.25	0.162 [−0.82, 6.79] d = 0.64
Left Knee	9.9 ± 3.5 (N = 25) [28]	-	14.89 ± 2.15	19.87 ± 7.48	0.672 [−0.21, 8.32] d = 0.22
Right Knee	17.75 ± 7.67 (N = 15) [14]	24.18 ± 9.21 (N = 20) [14]	10.18 ± 9.28	18.17 ± 8.81	0.048 * [1.11, 14.87] d = 0.81
Left Ankle	5.5 ± 3.0 (N = 10) [29]	-	8.43 ± 3.26	4.01 ± 4.54	0.001 * [2.04, 6.81] d = 1.29
Right Ankle	6.07 ± 2.90 (N = 15) [14]	5.73 ± 3.77 (N = 20) [14]	8.47 ± 4.19	4.13 ± 4.54	0.013 * [1.28, 7.39] d = 0.98

* denotes a statistically significant difference between the groups.

## Data Availability

Due to patient rights, data sharing is not permitted for this article.

## Abbreviations

The following abbreviations are used in this manuscript:

ADS	Adult Degenerative Scoliosis
IMU	Inertial Measurement Unit
EMG	Electromyography
RoM	Range of Motion
CoM	Center of Mass
MRI	Magnetic Resonance Imaging
BMI	Body Mass Index
DoF	Degree of Freedom
OMC	Optical Motion Capture
mm	Milimeter
RA	Right Ankle
LA	Left Ankle
SD	Standard Deviation
GRF	Ground Reaction Force
MOCAP	Motion Capture
